# A new sensitive automated assay for procalcitonin detection: LIAISON^®^ BRAHMS PCT^®^ II GEN

**DOI:** 10.1016/j.plabm.2016.06.002

**Published:** 2016-06-27

**Authors:** Antonio Fortunato

**Affiliations:** Laboratorio di Chimica Clinica ed Ematologia, Ospedale “San Bortolo”, Vicenza, Italy

**Keywords:** PCT, Bacterial infections, Sepsis, Antibiotic treatment, Automated diagnostic detection methods

## Abstract

**Objectives:**

To assess the performance of LIAISON^®^ BRAHMS PCT^®^ II GEN (DiaSorin, Saluggia, Italy) in procalcitonin (PCT) determination by comparing it to the assay reference method B·R·A·H·M·S PCT KRYPTOR (Thermo Fisher Scientific Clinical Diagnostics, Hennigsdorf, Germany) and assessing its ability to discriminate between healthy subjects and patients with suspected infection.

**Methods:**

Diagnostic performance was evaluated on: a) 193 selected samples covering the assay range, whose procalcitonin levels were already evaluated with the B·R·A·H·M·S PCT^®^ KRYPTOR; b) prospective samples: 150 apparently healthy specimens obtained from a blood bank, 161 hospitalized patients (not with specific pathologies), 243 apparently healthy children.

**Results:**

The comparison of LIAISON^®^ BRAHMS PCT^®^ II GEN to the reference method B·R·A·H·M·S PCT KRYPTOR yielded high correlation coefficients: slope of Deming fit equal to 1.04 (95% CI: 0.99–1.09) with an intercept equal to 0.05 (95% CI: −0.09 to 0.19) and a high concordance (98.4% (95% CI: 95.5–99.7%)) at the 0.5 ng/mL cut-off. Moreover, the results obtained using prospective samples showed: (i) no samples with PCT concentration >0.5 ng/mL (cut-off) for the apparently healthy adults (highest value=0.033 ng/mL, 95th percentile and 97.5th percentile <0.02 ng/mL); (ii) 2 samples >0.5 ng/mL for hospitalized patients (highest value=0.715 ng/mL, 95th percentile: 0.054 ng/mL; 97.5th percentile: 0.088 ng/mL); (iii) 3 samples >0.5 ng/mL for the healthy children population (highest value=0.713 ng/mL, 95th percentile: 0.155 ng/mL; 97.5th percentile: 0.275 ng/mL).

**Conclusion:**

The fully automated LIAISON^®^ BRAHMS PCT^®^ II GEN agrees well with the reference method and is suitable for early diagnosis of sepsis, severe bacterial infection and guiding antibiotic therapy.

## 1 Introduction

Over the last two decades, an increasing number of studies have evidenced the diagnostic and prognostic role of procalcitonin (PCT) in discriminating between bacterial, viral or other non-bacterial infection, sepsis and systemic inflammation [1] across a variety of clinical scenarios, in both adult and pediatric populations [2].

PCT concentrations increase at the onset of bacterial infection and correlate with the severity of infection. In healthy adults, plasma PCT concentrations generally remain below 0.05 ng/mL and rapidly increase within 2–4 h in case of systemic bacterial infection (<0.25 to <0.5 ng/mL), sepsis (>0.5 to >2 ng/mL) and some non-infectious conditions (including trauma, surgery, burns, hyperthermia and neoplasms), remaining high until recovery (Table 1) [3], [4], [5]. Slightly higher PCT levels are also found in neonates shortly after birth but gradually return to within normal range within 48 h [6], [7]. In all cases of inflammatory response, it is generally accepted that a continuous decline of PCT concentrations to >30% from peak values per day indicates significant improvement of systemic inflammation. Currently accepted cut-off reference values for PCT concentrations are summarized in Table 1.

**Table 1:** PCT concentrations cut-off reference values.

**PCT serum level**	**Reference value**
0.05 ng/mL	Healthy
<0.25–0.5 ng/mL	Systemic bacterial infection
0.5 ng/mL	Local infection
>0.5 to >2 ng/mL	Sepsis and non-infectious conditions
≥2 to ≤10 ng/mL	Severe sepsis
10 ng/mL	Sepsis shock

Such reference values must be considered together with individual patient's clinical conditions. Because PCT increase is associated with severity of systemic inflammatory response, low PCT values do not exclude for local bacterial infection, or viral infection. PCT levels can also be influenced by other non-infectious conditions such as severe trauma, autoimmune disorders, pancreatitis, renal dysfunction; hence reference ranges must be taken from current literature for specific condition or study population [adapted from [4], [5].

Specifically, PCT is ubiquitous and produced in the C cells (parafollicular cells) of the thyroid gland as a pro-hormone of calcitonin (CT) and cleaved by intracellular proteolytic enzymes into the active hormone [3]. It is released by parenchymal cells, including liver cells, kidney cells, adipocytes, and muscle cells in response to endotoxin or mediators released during bacterial infections (e.g. interleukin (IL)-1b, tumor necrosis factor (TNF)-a, and IL-6) [8]. Since PCT secretion into serum most likely derives from neuroendocrine cells in the lungs or intestine as part of the systemic inflammatory response, it has been termed a “hormokine” [9], [10], [11]. In contrast to cytokines, PCT release is more tightly controlled and found in the systemic circulation alone during response to severe stress and sepsis [4], [12], [13]. During homeostasis, PCT circulates at very low levels, whereas in bacterial infections, serum PCT levels start to rise at 4 h after the onset of systemic infection, and peak at 6 h, maintaining a plateau between 8 and 24 h [14]. Circulating PCT levels halve daily when the infection is controlled by the host immune system or antibiotic therapy [15].

Due to its peculiar biologic profile and “hormone-kine like” behavior PCT has been demonstrated to be a much more refined and reliable indicator of infection than other markers currently available, featuring a higher sensitivity and specificity to other markers such as such as C-reactive protein (CRP), cytokines (IL-6, IL-8, IL-10) and lactate [9], [16], [17], [18], [19], [20].

In addition to being used as a diagnostic marker, the measurement of PCT concentration is increasingly being used as part of antimicrobial stewardship activities to monitor progression of infection and to begin/suspend antibiotic therapy [21], [22].

To date, several commercial quantitative and qualitative PCT assays are available with different detection limits. The aim of the present study was to analyze the discrimination ability of the LIAISON^®^ BRAHMS PCT^®^ II GEN (DiaSorin, Saluggia, Italy) in healthy subjects and patients with suspected infection and compare the diagnostic performance of LIAISON^®^ BRAHMS PCT^®^ II GEN to the reference method B·R·A·H·M·S PCT^®^ KRYPTOR (Thermo Fisher Scientific Clinical Diagnostics, Hennigsdorf, Germany).

## 2 Patients and methods

### 2.1 Study population

A total of 193 samples obtained from patients hospitalized at Vicenza Hospital (Italy) in September 2014 and previously tested with the reference method B·R·A·H·M·S PCT^®^ KRYPTOR, were tested with LIAISON® BRAHMS PCT® II GEN. They were 112 men with a mean age of 67 years and 81 women with a mean age of 64 years. To validate the sensitivity and specificity of the LIAISON® BRAHMS PCT® II GEN method, a population of adults and children were prospectively analysed: 311 adult patients and 243 children were enrolled. Out of 311 adult specimens, 150 were collected from apparently healthy subjects from a European blood bank and 161 were hospitalized patients from the Chivasso Hospital (Italy). Baseline and demographic patients data are not available. A group of 243 healthy outpatient children, 122 boys and 121 girls aged 1–12 years, with no clinical or biological sign of infection and no evidence of endocrine disorders, from microbiology laboratories of the Area Vasta Romagna (AVR, Italy) were enrolled. Subjects positive for IgM or monitored for maternal infection were excluded. These samples were collected to ensure that both genders and two age classes (1–6 years and 7–12 years) were represented adequately.

### 2.2 Sample handling

PCT quantification was carried out on both serum or plasma. The manufacturer has confirmed equivalence between serum, lithium heparin plasma, citrate plasma and EDTA plasma [23]. We measured the PCT levels in sera of the both healthy population (adults and children) and in plasma of the 193 selected samples. Specifically, the 193 samples from Vicenza Hospital (Italy) were collected in lithium-heparin tubes, centrifuged and separated; they were stored at −18 °C, then thawed and tested with the reference method (B·R·A·H·M·S PCT^®^ KRYPTOR) and with LIAISON^®^ BRAHMS PCT^®^ II GEN. The samples from healthy children (243 samples, Area Vasta Romagna), those from healthy adults (150 samples from blood bank) and those from hospitalized adults (161 samples, Chivasso Hospital) were collected in serum tubes, centrifuged and separated, stored at −18 °C, then unfrozen and tested with LIAISON^®^ BRAHMS PCT^®^ II GEN.

### 2.3 PCT measurement

For the 193 selected samples, LIAISON^®^ BRAHMS PCT^®^ II GEN determinations were compared with the concentrations obtained from the B·R·A·H·M·S PCT^®^ KRYPTOR assay, considered the reference assay in most studies for determining PCT levels in plasma.

Both PCT methods are automated homogeneous sandwich immunoassay based on antibodies against the CP and katacalcin domains (CCP-I) of the prohormone: in particular, they use a sheep polyclonal anti-calcitonin antibody and a monoclonal anti-katacalcin antibody, whereby the PCT molecules are bound by these 2 antibodies.

Briefly, the B·R·A·H·M·S PCT KRYPTOR assay is based on TRACE technology (time-resolved amplified cryptate emission), while the LIAISON^®^ BRAHMS PCT^®^ II GEN system is a chemiluminescence immunoassay (CLIA) technology with paramagnetic microparticle solid phase.

Essentially, TRACE technology relies on measurement of the nonradioactive energy transfer from a donor molecule to an acceptor molecule. In the KRYPTOR assay, the donor molecule is a europium cryptate-labeled polyclonal sheep antibody that recognizes epitopes in the immature CP region of PCT, and the acceptor molecule is an XL665-labeled monoclonal antibody against the CCP-I region of PCT. If PCT is present in the sample, it is sandwiched between the two molecules forming an immunocomplex enabling a transfer of energy between the 2 tracers, donor and acceptor molecules, accompanied by an amplification.

In the LIAISON^®^ BRAHMS PCT^®^ II GEN system the two different highly specific monoclonal antibodies are used for the coating of the solid phase (magnetic particles, mouse monoclonal anti- katacalcin antibody) and for the tracer, labeled with isoluminol, mouse monoclonal anti-calcitonin antibody. The intensity of the signal, and hence the amount of isoluminol-antibody conjugate, is measured by a photomultiplier as relative light units (RLU).

The manufacturer claims intra-assay coefficients of variation (CV)<5% and inter-lot CVs between 4.4% and 17.6%.

In addition, the manufacturer claims that both assays are unaffected by human calcitonin and katacalcin levels, even at high concentrations; no cross-reactions were observed [23]. The assay performance is also claimed not to be influenced by conjugated and unconjugated bilirubin, hemoglobin or triglycerides [23].

The main characteristics of the two assays are summarized in Table 2. Specifically, the KRYPTOR method has a measuring range of 0.02–1000 ng/mL, a functional sensitivity (20% total CV) of 0.06 ng/mL and a time to the first result of 19 min. Whereas, the time for the first result of the LIAISON^®^ BRAHMS PCT^®^ II GEN method is 16 min with a measuring range of 0.02–100 ng/mL and a functional sensitivity of 0.04 ng/mL.

**Table 2:** Main characteristics of assays BRAHMS PCT^®^ KRYPTOR and LIAISON^®^ BRAHMS PCT^®^ II GEN.

**Property**	**BRAHMS PCT^®^ KRYPTOR**	**LIAISON® BRAHMS PCT^®^ II GEN**
Sample volume	50 μl	100 μl
Time for the first result	19 min	16 min
Measuring range	0.02–1000 ng/mL	0.02–100 ng/mL
Calibration	1 point	2 point
Calibration stability	7 days	8 weeks
Integral on board stability	14 days	12 weeks
Functional assay sensitivity	0.06 ng/mL	0.04 ng/mL
LoD	0.02 ng/mL	0.02 ng/mL
Matrix	Serum/plasma	Serum/plasma

Functional sensitivity of the assay, described also as the Limit of Quantification (LoQ), is defined as the lowest analyte concentration that can be determined with an inter-assay coefficient of variation (CV)<20%; the Limit of Detection (LoD), defined also as analytical sensitivity, is the lowest analyte concentration likely to be reliably distinguished from the Limit of Blank (LoB) (the highest apparent analyte concentration expected to be found when replicates of a blank sample containing no analyte are tested) and at which detection is feasible; thus LoD is determined by utilizing both the measured LoB and test replicates of a sample known to contain a low concentration of analyte. These factors have been established following the Clinical and Laboratory Standards Institute (CLSI) guideline EP17-A [24].

All assay methods are based on an internal master curve with 1-point and 2-point calibration, respectively for KRYPTOR and LIAISON^®^ BRAHMS PCT^®^ II GEN assay. As a point of reference, a healthy individual's baseline PCT level, typically of 0.1 mg/mL, is used.

### 2.4 Statistical analyses

The analyses were carried out using Deming regression analyses, Spearman's correlation coefficient and Bland-Altman difference plot to compare the two different measurement methods, and agreement at the clinically relevant cut-off was calculated with kappa statistic. The level of statistical significance was set at p≤0.05. As a useful complementary plot to Bland–Altman, a mountain plot was also performed. This plot allows the comparison of 2 or 3 laboratory assays. A mountain plot (or “folded empirical cumulative distribution plot”) is created by computing a percentile for each ranked difference between a new method and a reference method. To obtain the plot, the following transformation is performed for all percentiles above 50: percentile=100−percentile. These percentiles are then plotted against the differences between the two methods [25]. The mountain plot was used because it makes it easier to find the central 95% of the data, even when the data are not normally distributed.

## 3 Results

The defined cut-off for exclusion of bacterial infections and sepsis is the PCT concentration of ≤0.2 ng/mL [4]. High PCT levels have a high positive predictive value to rule in the diagnosis of sepsis (PCT >0.5 to >2 ng/mL), whereas normal or very low PCT plasma concentrations have a high negative predictive value to rule out severe systemic inflammation or sepsis (PCT <0.25 to <0.5 ng/mL).

The diagnostic performance of LIAISON^®^ BRAHMS PCT^®^ in measuring PCT level was assessed in comparison with the reference method.

**Fig. 1:**
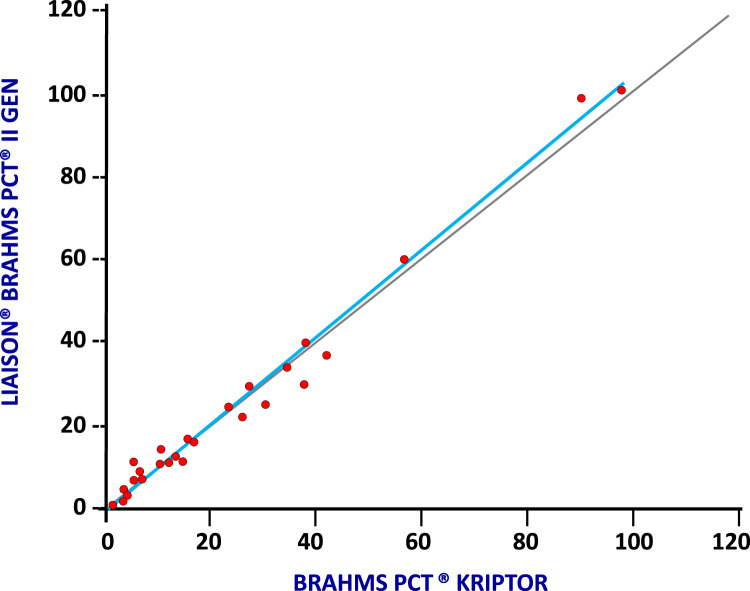
BRAHMS PCT^®^ KRYPTOR vs LIAISON^®^ BRAHMS PCT^®^ II GEN correlation (r=0.99).

PCT levels were measured both with LIAISON^®^ BRAHMS PCT^®^ II GEN and B·R·A·H·M·S PCT^®^ KRYPTOR reference method in 193 selected samples, covering the assay reading range, in order to compare the two different assays. As shown in Fig. 1, an excellent correlation (correlation coefficient, r=0.99, obtained by linear regression analysis) both in term of slope and intercept was obtained: a slope of Deming fit equal to 1.04 (95% CI: 0.99–1.09) with an intercept of 0.05 (95% CI: −0.09 to 0.19) (Fig. 1).

**Fig. 2:**
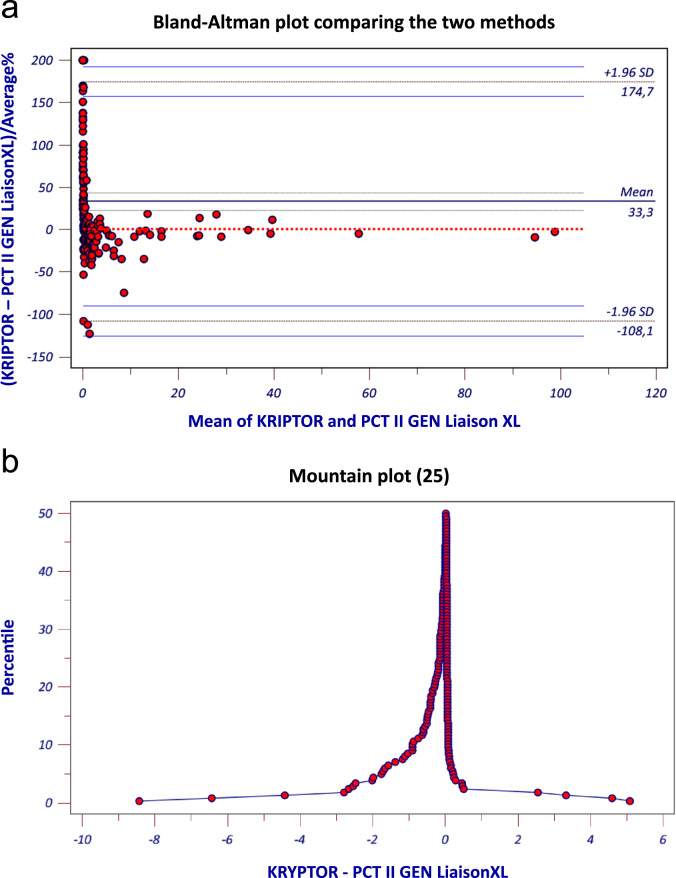
(a) Bland-Altman plot comparing the two methods; and (b) mountain plot [25].

The graphs in Fig. 2 show that there are very small differences at very low concentrations which are of no clinical significance.

A good correlation between the two methods was observed also in terms of clinical classification as indicated in Table 3. In particular, the percentage of concordance between the two assays using a cut off of 0.5 ng/mL is 98.4% (95% CI: 95.5–99.7%) as reported in Table 4.

**Table 3:** Comparison of the two PCT measuring methods using a cut off of 0.5 ng/mL.

		**BRAHMS PCT^®^ KRYPTOR**		**Total**
		**<0.5 ng/mL**	**≥0.5 ng/mL**	
**LIAISON® BRAHMS PCT ® II GEN**	**<0.5 ng/mL**	101	1	102
	**≥0.5 ng/mL**	2	89	91
	**Total**	103	90	193

**Table 4:** Classification concordance between LIAISON^®^ BRAHMS PCT^®^ II GEN and B·R·A·H·M·S PCT^®^ KRYPTOR.

	**Agreement (%)**	**95% CI (%)**
**<0.5 ng/mL**	98.1 (101/103)	93.2–99.8
**≥0.5 ng/mL**	98.9 (89/90)	94–100
**Total**	98.4 (190/193)	95.5–99.7

To assess the utility and clinical value of LIAISON^®^ BRAHMS PCT^®^ II GEN in measuring PCT for diagnosis and to evaluate its performance in patients without apparent infection, prospective samples from 311 adults and 243 children were analysed. Since PCT diagnostic indications in children are similar to those for adults, the pediatric population was enrolled with the aim to implement and corroborate the specificity of the method utilized. The results are summarized in Table 4: none of the samples of the 150 apparently healthy adult population showed a PCT concentration >0.5 ng/mL cut-off value—the highest PCT value reported being equal to 0.033 ng/mL (95th percentile as well as 97.5th percentile lower than 0.02 ng/mL); only 2 samples among the 161 hospitalized patients exhibited PCT concentration >0.5 ng/mL— with the highest value being 0.715 ng/mL (95th percentile: 0.054 ng/mL; 97.5th percentile: 0.088 ng/mL); and 3 samples of the healthy children population, reported a PCT concentration over the cut-off, with the highest value equal to 0.713 ng/mL (95th percentile: 0.155 ng/mL; 97.5th percentile: 0.275 ng/mL).

Despite the lack of a counterpart hospitalized children population, we selected this pediatric population, with no clinical or biological sign of infection, to emphasize the performance of the LIAISON^®^ BRAHMS PCT^®^ II GEN in evaluating normal PCT levels. As reported in Table 5, only 5 samples from this population showed a PCT concentration >0.5 ng/mL. Specifically, 3 samples among the healthy children population and 2 samples in hospitalized patients' population resulted over 0.5 ng/mL. These samples were re-tested using Vidas Brahms PCT®, with comparable results (Table 6).

**Table 5:** PCT measurement in healthy and hospitalized study patients prospectively analysed by LIAISON^®^ BRAHMS PCT^®^ II GEN indicating 95th and 97.5th centiles of the distribution.

**Subjects**	**N**	**Mean dose (ng/mL)**	**95th (ng/mL)**	**97.5th (ng/mL)**	**>0.5 ng/mL**
Healthy adults	150	<0.020	<0.020	<0.020	0
Healthy children	243	<0.020	0.155	0.275	3
Hospitalized adults	161	<0.020	0.054	0.088	2

**Table 6:** Samples with PCT concentration >0.5 ng/mL re-tested using Vidas Brahms PCT®.

**ID**	**LIAISON^®^ Brahms (ng/mL)**	**VIDAS Brahms (ng/mL)**
Hospitalized 1	0.69	0.57
Hospitalized 2	0.56	0.52
Children 1	0.50	0.46
Children 2	0.71	0.60
Children 3	0.55	0.53

## 4 Discussion

Currently, there are several different assays available for PCT measurement. This study compared the performance of the LIAISON^®^ BRAHMS PCT^®^ II GEN system, a chemiluminescence immunoassay (CLIA) technology, with the reference method BRAHMS PCT^®^ KRYPTOR assay which is based on TRACE technology, and highlights the quality of LIAISON^®^ BRAHMS PCT^®^ II GEN system in terms of sensitivity and specificity, both in adults and children populations.

The LIAISON^®^ BRAHMS PCT^®^ II GEN assay showed a good correlation and concordance with the reference method, which is highly relevant for the routine implementation of PCT. In addition, this implies that switching from one assay to the other should not have a clinically relevant influence on comparing data between the LIAISON^®^ and the KRYPTOR assay.

The LIAISON^®^ BRAHMS PCT^®^ II GEN assay could also represent a key component for PCT-guided antibiotic therapy and for use in the early diagnosis of severe bacterial respiratory tract infection, offering a fast, precise and reliable measurements of PCT. However, as for the other commercially available PCT measurement assays, results must be interpreted carefully in the context of medical history, physical examination and microbiological assessment, because increased PCT levels may not always be related to infection, and low PCT levels do not automatically exclude the presence of bacterial infection. Therefore, clinicians should use the PCT results in conjunction with the patient's other laboratory findings and clinical signs.

In summary, our results, although derived from relatively small groups of patients, confirm the utility of LIAISON^®^ BRAHMS PCT^®^ II GEN assay as a sensitive method for PCT determination.

## Disclosures

The present study did not rely on any external funding sources.
